# Anaplastic carcinoma in ovarian seromucinous cystic tumor of borderline malignancy

**DOI:** 10.1186/s13048-018-0449-1

**Published:** 2018-09-03

**Authors:** Toshiyuki Okumura, Etuo Muronosono, Masahiko Tsubuku, Yasuhisa Terao, Satoru Takeda, Masanori Maruyama

**Affiliations:** 1 Department of Obstetrics and Gynecology, Maruyama Memorial General Hospital, 2-10-5 Motomachi, Iwatukiku, Saitamashi, Saitama, 339-8521 Japan; 2 Department of Radiology, Maruyama Memorial General Hospital, Saitama, Japan; 3 Department of Obstetrics and Gynecology, Juntendo University School of Medicine, Tokyo, Japan

**Keywords:** Ovarian tumor, Seromucinous borderline malignancy, Mural nodule, Anaplastic tumor, Immunohistochemical, Case report

## Abstract

**Background:**

The mortality rate of ovarian cancer is the highest among all gynecological malignancies in Japan. Ovarian tumors are classified as benign, borderline malignant, or malignant. Anticipating the histological subtype with imaging only is often difficult because of several histological subtypes of epithelial ovarian tumors (such as serous, mucinous, endometrioid, clear cell, and Brenner tumors). In addition, the majority of mucinous tumors in the ovary are metastatic. Furthermore, mucinous tumors belong to one of the two different subclasses (i.e., intestinal and seromucinous types). Ovarian seromucinous cystic tumors of borderline malignancy are infrequent and only rarely coexist with other malignant tumors.

**Case presentation:**

We have reported a 53-year-old Japanese woman with anaplastic carcinoma in an ovarian seromucinous cystic tumor of borderline malignancy. Her MRI and CT analysis revealed an ovarian tumor with a mural nodule, ascites, and peritoneal dissemination. Enhanced MRI revealed that the mural nodule was enhanced. Enhanced CT analysis revealed that the lymph nodes were not swollen. Intriguingly, the mural nodule crossed the cyst wall into the cavity and onto the surface. Her laboratory data revealed high serum CA 125 level. Cumulatively, these results suggested ovarian malignancy. The patient underwent hysterectomy with bilateral salpingo-oophorectomy, omentectomy, and resection of the disseminated lesions. Lymph node biopsy was omitted because of the suggestion of enhanced CT image findings and palpation during surgery. Her postoperative specimen examination determined FIGO at least stage IIIB, and accordingly, adjuvant chemotherapy was prescribed. After 3 years of the operation, the patient is presently alive without clinical tumor recurrences.

**Conclusion:**

Imaging studies with pathognomonic findings contributed to ovarian cancer diagnosis in this case. To the best of our knowledge, this is the first study in English literature to report detailed classification of mucinous borderline malignancy, seromucinous cystic, and anaplastic carcinoma in an ovarian seromucinous cystic tumor of borderline malignancy.

## Background

**Fig. 1:**
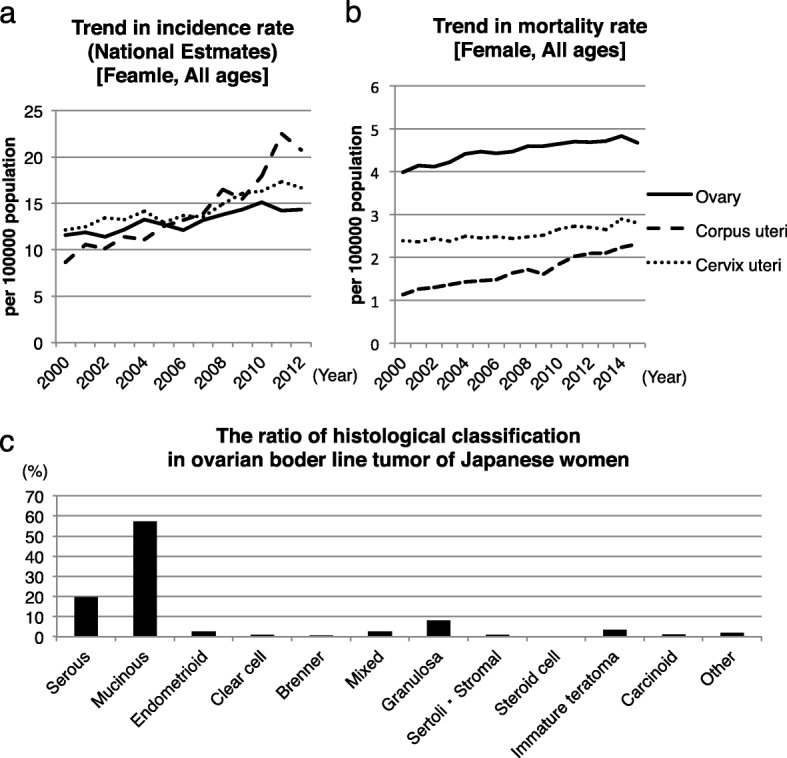
Statistical analysis. **a** Gynecological malignancy incidence rate in Japanese women by year. **b** Gynecological malignancy mortality rate in Japanese women by year. **c** Percentages of ovarian borderline malignancies in Japanese women by histological classification

Ovarian tumors are classified as benign, borderline malignant, or malignant. The mortality rate of ovarian malignancies is the highest among all gynecological malignancies (Fig. 1a, b) [1]. In general, ovarian malignancies display various histological types. The ovarian mucinous borderline malignancies represent 58% and the serous borderline malignancies represent 19% of all borderline malignancies in Japan (Fig. 1c). In contrast, these percent values are 42% and 52.1% of the borderline malignancies, respectively, in the Western countries [2, 3].

Ovarian mucinous borderline malignancies used to be classified earlier into intestinal and endocervical types. However, the World Health Organization changed their name from endocervical to seromucinous type in 2014 [4]. Nakashima reported that ovarian mucinous borderline malignancy in Japan encompasses 38% of intestinal, 36% of seromucinous, and 26% of mixed types [5]. However, Prat reported that 90% of ovarian mucinous borderline malignancies in the Western countries are of the intestinal type [6].One of the most common ovarian epithelial cancer considered to demonstrate racial group differences is ovarian clear cell carcinoma. Clear cell carcinoma was significantly more prevalent among Asian women (12%) than among any other race (5%) as per the SEER data in the United States [7]. Endometriosis is considered as one of the risk factor associated with clear cell carcinoma [8]. However, the available literature negated that the prevalence of endometriosis is high among Asian women than in other races [9]. High adiposity is reported as a risk factor of ovarian cancer in African–American women [10]. Furthermore, oral contraception, parity, and breastfeeding has been reported to decrease the risk of ovarian cancer [11]. In reference to ovarian borderline malignancy, the incidence rate is high in white women than in black women in the United States [12]. In consideration of these literatures, the difference between the incidence rates for ovarian borderline malignancy among different races may be attributed to the feeding habit, antifertility method used, and economic situation. Furthermore, the genetic differences between races possibly influence the incidence rate of ovarian borderline malignancy. Hence, we need to collect more data and perform further studies to clarify this possibility. Scully reported 7 cases of ovarian mucinous tumors with mural nodules in 1979 [13]. Mural nodules are classified into 6 groups: anaplastic carcinoma, sarcoma, carcinosarcoma, sarcoma-like nodule, mixed nodule, and smooth muscle sarcoma [14]. Some case studies have reported that ovarian seromucinous borderline malignancies can coexist with secondary tumors such as endometrioid carcinoma, clear cell carcinoma, or squamous cell carcinoma [15–17]. Indeed, a case of ovarian mucinous borderline malignancy coexisting with anaplastic carcinoma has been reported previously [18]. However, this previous report did not include data on tumor subtyping. To the best of our knowledge, our paper is the first report of an ovarian seromucinous borderline malignancy coexisting with an anaplastic carcinoma.

## Case presentation

**Fig. 2:**
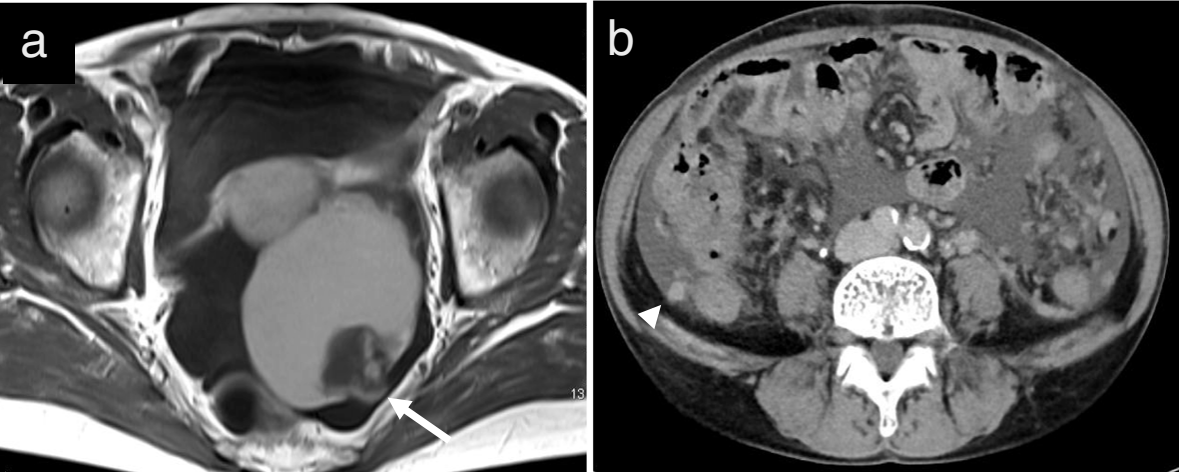
Image analyses. **a** Gadorinium-enhanced T1-weighted magnetic resonance imaging showing a mural nodule that was enhanced and the mural nodule crossed the cyst wall into the cavity and onto the surface (arrow). **b** Enhanced computed tomography showing ascites in the pelvis and intraperitoneal malignant dissemination (arrowhead)

**Fig. 3:**
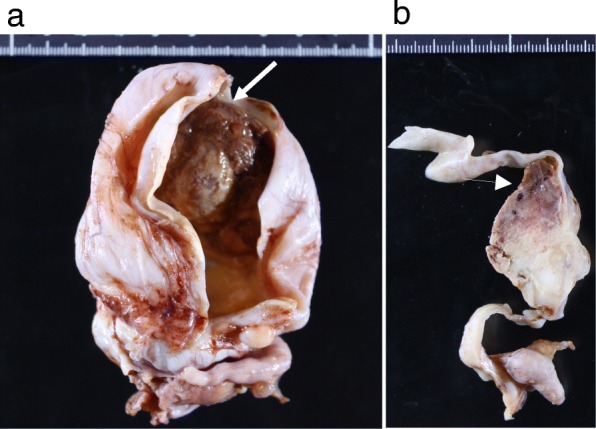
Gross findings of isolated preparations. **a** Chocolate-like cyst contents. Medial and lateral mural nodule enlargement (arrow). **b** Brown color of a part of the mural nodule (arrow head)

**Fig. 4:**
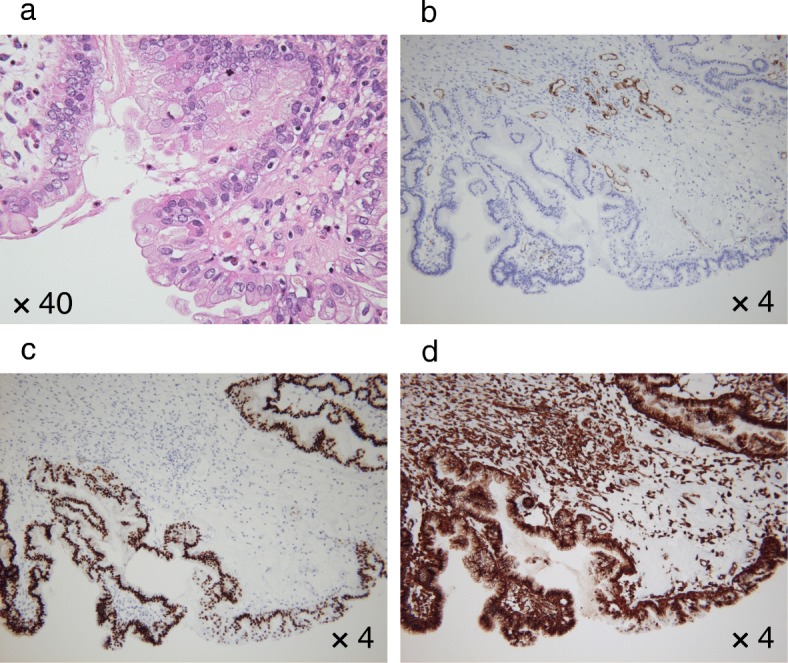
Microscopic and immunohistochemical cyst wall findings. **a** Hematoxylin and eosin-stained section showing epithelium with papillary hyperplasia in the cyst wall. The epithelium is mucinous and shows dysplasia. **b** Cyst wall showing negative immunohistochemical staining for WT-1. **c** Cyst wall showing positive immunohistochemical staining for estrogen receptor. **d** Cyst wall showing positive immunohistochemical staining for vimentin

**Fig. 5:**
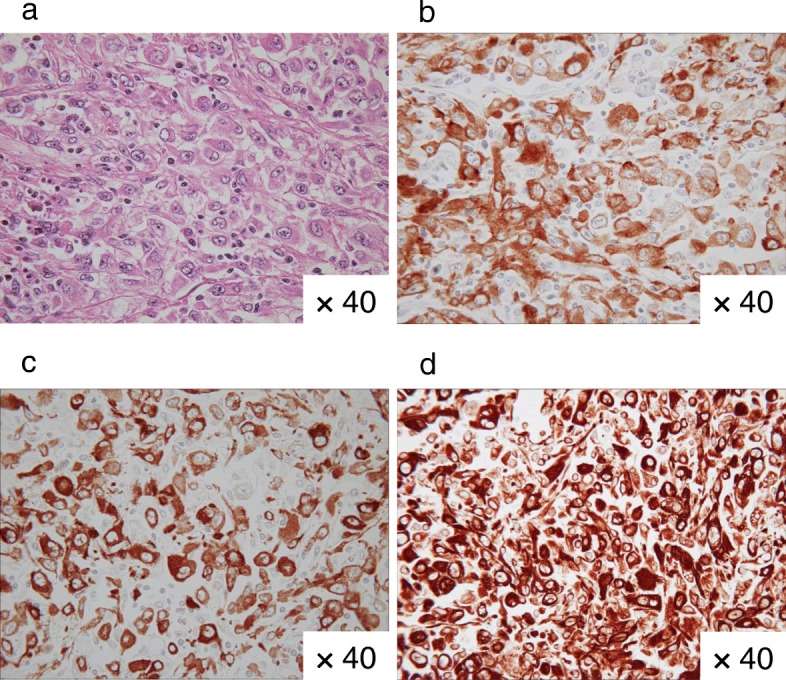
Microscopic and immunohistochemical mural nodule findings. **a** Hematoxylin and eosin-stained section showing dense, undifferentiated, polymorphic, and eosinophilic cells with hyperplasia in the mural nodule. **b** Mural nodule showing positive immunohistochemical staining for CAM5.2. **c** Mural nodule shows positive immunohistochemical staining for AE1/AE3. **d** Mural nodule showing positive immunohistochemical staining for vimentin

A 53-years-old Japanese woman with ascites and a pelvic tumor was transferred to the Maruyama Memorial General Hospital on the suspicion of ovarian malignancy. A trans-vaginal ultrasound examination confirmed a large ascites volume and approximately 10-cm single ovarian cystic tumor with a mural nodule. The serum level of CA19–9 was 37 IU/L and that of CA125 was increased markedly at 333 IU/L. Magnetic resonance imaging (MRI) revealed ascites and an approximately 10-cm single cystic tumor with a 4-cm mural nodule. T1- and T2-weighted MRI revealed a high-intensity cystic area. In addition, the T1-weighted and fat-suppressed MRI showed the high-intensity cystic area. Therefore, we expected the cyst to contain blood. Moreover, gadorinium-enhanced T1-weighted MRI revealed an enhanced mural nodule (Fig. 2a). Interestingly, the mural nodule crossed the cyst wall into the cavity and onto the surface, which is an extremely rare finding and may be the characteristic of this tumor type. Computed tomography (CT) imaging showed a large ascites volume and tumor dissemination throughout the pelvis (Fig. 2b). On the basis of these examinations, we suspected a malignant tumor. The patient underwent bilateral salpingo-oophorectomy with hysterectomy, omentectomy, resection of disseminated lesions and optimal debulking of the tumor. Lymph node biopsy was omitted because of the suggestion of enhanced CT image findings and palpation during surgery. The content of the left ovarian cyst was chocolate-like. We identified a mural nodule of approximately 4-cm size on the cyst wall (Fig. 3a, b). The histopathological examination revealed columnar tumor cells on the cyst wall. Eosinophilic epithelial cells suggestive of papillary hyperplasia and squamous metaplasia were identified on the endometriosis tissues. The cells showing dysplasia were categorized as borderline malignant. The cyst showed positivity for estrogen receptor and vimentin antibodies, and it was negative for WT-1; therefore, the immunohistochemical staining led to the diagnosis of ovarian seromucinous borderline malignancy (Fig. 4a–e). The mural nodule contained dense hyperplastic polymorphic and eosinophilic undifferentiated cells. The mural nodule was positive for CAM5.2, AE1/AE3, and vimentin and was identified as an anaplastic carcinoma by immunohistochemistry (Fig. 5a–e). A nodule present in the omentum showed the same pathological findings as the mural nodule and was thus diagnosed as a metastasis from the anaplastic carcinoma. The disseminated lesion was < 2 cm in size. We classified the ascites as class I by cytodiagonosis and diagnosed the patient as having an anaplastic carcinoma in the left ovarian seromucinous cystic tumor of borderline malignancy, with a FIGO stage IIIB. She underwent 6 courses of pacritaxel and carboplatin. After 3 years, the patient is still alive without any clinical findings of tumor recurrence.

## Discussion

Ovarian tumors are classified as benign, borderline malignant, or malignant. The most common borderline malignant ovarian tumors are serous and mucinous. Mucinous borderline malignancies can be further subclassified into either intestinal or seromucinous types. Fox et al. reported the first seromucinous-type tumor [19]. Malignancy and recurrences determine the prognosis of seromucinous borderline malignancy tumors [20]. Endometriosis is a pathognomonic sign of approximately 30% of seromucinous type tumors [21]. Approximately 33% of all seromucinous-type tumors present with a mutation in the tumor-suppressor gene *ARID1A*; therefore, it can be considered as one of the responsible genetic factors [22]. Moreover, *ARID1A* is believed to be involved in the pathogenesis of endometriosis. In fact, 46% of ovarian clear cell carcinomas and 30% of ovarian endometrioid carcinomas have *ARID1A* mutations [23]. Mesbah Ardakani et al. compared the molecular profiling of ovarian mucinous tumors with their matched mural carcinomatous nodules by next generation sequencing. They reported a case of *KRAS* mutation in the mural nodule and paired mucinous borderline malignancy. However, *p53* mutation was present in the mural nodule but not in mucinous borderline malignancy [24]. *p53* is one of the common tumor-suppressor genes. *p53* mutation is the most common and frequent event in high-grade serous ovarian carcinoma [25]. Mackenzie et al. reported that *p53* mutation occur more frequently in mucinous carcinoma than in mucinous borderline malignancy [26]. Thus, based on these reports, *p53* mutation possibly plays a vital role in the progression of mucinous ovarian tumors. A pathognomonic histological sign of seromucinous borderline malignancies is 100% estrogen receptor positivity by immunohistochemistry; in addition, 65% of cases show progesterone receptor positivity, 92% present show CA125 positivity, and 8% show WT-1 positivity [27]. In our case, we found that the pathological examination of endometriosis was compatible with the results of seromucinous border malignancy after immunohistochemical staining. Mural nodules require differentiation between anaplastic and sarcomatous tumors. Vimentin and CAM5.2 are usually positive in anaplastic tumors by immunohistochemical staining. However, sarcomatous tumors can yield the same immunohistochemistry result. AE1/AE3 can be used to discriminate between these subtypes because it is positive only in anaplastic tumors [28]. In the present case, mural nodule was positive for AE1/AE3; therefore, our patient was diagnosed with anaplastic tumor. Anaplastic tumors occurring in various organs, such as lung, pancreas, kidney, and thyroid, have high malignancy grade. Anaplastic tumor did not involve the prognosis of stage IA seromucinous borderline malignancies [29]. The differential diagnosis between an anaplastic tumor and a sarcomatous tumor is important for their proper treatment, because each tumor has a different prognosis [30]. In summary, we treated an extremely rare case of anaplastic tumor occurring in an ovarian seromucinous borderline malignant cyst. Although the anaplastic tumor had disseminated to the omentum, the conventional ovarian cancer chemotherapy administered to our patient resulted in recurrence-free survival of at least 3 years. To the best of our knowledge, this is the first English language report of an anaplastic tumor occurring in a seromucinous borderline malignancy ovarian cyst. Our study images show that the nodule enlargement in the medial and lateral directions are unusual and may represent a characteristic finding that is useful for the diagnosis of these types of tumor. Finally, our experience suggests that conventional chemotherapy is possibly effective in treating these tumors. However, we need to study more relevant cases to investigate whether conventional chemotherapy is effective.

## Conclusion

This is an extremely rare case that suggests that mural nodule of anaplastic tumor possibly has a characteristic finding from the study of imaging that the mural nodule crossed the cyst wall into the cavity and onto the surface. To the best of our knowledge, this is the first English language report of an anaplastic tumor occurring in a seromucinous borderline malignancy ovarian cyst.

## Abbreviations

ARID1A: AT-Rich Interaction Domain 1A
